# BTF3 sustains cancer stem-like phenotype of prostate cancer via stabilization of BMI1

**DOI:** 10.1186/s13046-019-1222-z

**Published:** 2019-05-28

**Authors:** Jing Hu, Feifei Sun, Weiwen Chen, Jing Zhang, Tao Zhang, Mei Qi, Tingting Feng, Hui Liu, Xinjun Li, Yuanxin Xing, Xueting Xiong, Benkang Shi, Gengyin Zhou, Bo Han

**Affiliations:** 1 The Key Laboratory of Experimental Teratology, Ministry of Education and Department of Pathology, School of Basic Medical Sciences, Shandong University, Jinan, 250012 China; 2 Department of Biochemistry and Molecular Biology, School of Basic Medical Science, Jinan, 250012 China; 3 Department of Pharmacy, Shandong Provincial Hospital Affiliated To Shandong University, Jinan, 250021 China; 4 Department of Epidemiology and Biostatistics, School of Public Health, Shandong University, Jinan, 250012 China; 5 Department of Pathology, Shandong University QiLu hospital, Jinan, 250012 China; 6 Department of Pathology, Binzhou People’s Hospital, Binzhou, 256610 China; 7 Department of Molecular Genetics, University of Toronto, M5S1A8, Toronto, ON Canada; 8 Department of Urology, Shandong University QiLu hospital, Jinan, 250012 China

**Keywords:** Prostate cancer, Cancer stem-like traits, BTF3, BMI1

## Abstract

**Background:**

Cancer stem-like traits contribute to prostate cancer (PCa) progression and metastasis. Deciphering the novel molecular mechanisms underlying stem-like traits may provide important insight for developing novel therapeutics.

**Methods:**

Immunohistochemistry and immunofluorescence assays in prostatic tissues; gain- and loss-of-function analyses using ectopic overexpression and shRNAs in PCa cell lines; measurements of tumorigenic and stemness properties, and transcription in vitro and in vivo; transcriptional analysis in public databases.

**Results:**

We identified that overexpression of BTF3 in PCa tissues and BTF3 expression highly correlates to stem-like traits. Cancer stem-like characteristics in PCa including self-renewal and metastatic potential were impaired by BTF3 loss and promoted by BTF3 overexpression. Mechanistically, BTF3 could stabilize BMI1, which is a crucial regulator of prostate stem cell self-renewal. More importantly, our data revealed that BTF3 is highly predictive of poor prognosis and may help in risk stratification of PCa patients.

**Conclusions:**

BTF3 promotes PCa progression though modeling stem-like traits in PCa. BTF3 represents a stratification marker in PCa progression and outcomes.

**Electronic supplementary material:**

The online version of this article (10.1186/s13046-019-1222-z) contains supplementary material, which is available to authorized users.

## Background

Prostate cancer (PCa) is the second leading malignancy in males and the fourth most common tumor type worldwide [1]. There is increasing evidence that cancer stem cells (CSCs) are likely the main cause of tumor initiation, metastasis, recurrence and intrinsic resistance of standard treatment in most human cancers, including PCa [2–4]. Certain PCa cells with stem-like traits generate more aggressive tumor and survive androgen ablation therapy [5]. Characterizing the novel molecular mechanisms underlying PCa progression, especially those regulating the stem-like traits, may provide crucial insight for developing novel therapeutics.

The unique ability of embryonic stem cells (ESCs) and adult tissue stem cells (ASCs) to self-renew and give rise to multiple cell lineages have formed the basic definitions of “stemness” [6]. Gene sets that are enriched in ESCs and ASCs were frequently overexpressed in poorly differentiated tumors and were associated with poor clinical outcomes [6, 7]. Genes defining ESCs and prostate stem cells (PrSCs) (*SOX2*, *MYC, BMI1* etc) contribute to stem-like traits and aggressive phenotypes in PCa [8–10].

BMI1 is the core component of polycomb repressive complex 1, which functions to modulates transcription patterns epigenetically in development, stem cell maintenance, and differentiation [11]. Previous studies have demonstrated that BMI1 plays an important role in basal PrSCs maintenance as well as PCa initiation and progression [10, 12]. BMI1 is necessary for Hedgehog [13], Wnt signaling [14], and Akt-mediated self-renewal [3]. Targeting BMI1 in tumor initiating cells could be an effective strategy for PCa treatment [12, 15].

Basic transcription factor 3 (BTF3) is a 27 kDa protein that was first identified as a transcriptional factor that forms complex with RNA polymerase II [16, 17]. Subsequent studies revealed that BTF3 is also involved in protein regulation during translation and is therefore also known as Nascent- polypeptide associated complex β-subunit (βNAC) [18]. BTF3 is a conserved protein that plays an important role in the correct folding and the prevention of misfolding and aggregation of polypeptide chains [18]. It is well documented that BTF3 expression is vital in embryonic development; mutations or deletions of the BTF3 gene in mice, *Drosophila*, and *C. elegans* lead to the death of embryos at early stage of the development [19–21]. In addition, overexpression of BTF3 has been shown to be associated with a variety of malignancies, including cancer of the pancreas, colon, stomach, prostate and breast [22–26]. Wang et al. defined an ESC-like transcriptional program involving overexpression of *BTF3* in both human and mouse ESCs as well as embryonal carcinoma cells. The ESC signature is activated in diverse human epithelial cancers and strongly predicts metastasis and death [6]. Inhibition of BTF3 reduces the proliferative and metastatic capacity, and sensitizes luminal breast cancer cells to PI3Kα inhibitors [26]. However, the mechanisms of BTF3 in cancer progression remain unclear.

In this study, we demonstrate that downregulation of BTF3 impairs the stem-like traits of PCa cells, and thus their malignant behaviors. Mechanically, we show that BTF3 stabilizes BMI1 by blocking proteasome-mediated degradation. Clinically, BTF3 is overexpressed in a subset of PCa patients with stem-like traits and poor prognosis.

## Methods

### Patients and tissue microarrays

Four tissue microarrays were constructed for 315 PCa cases using 1.0 mm cores as previously described [27]. The first cohort consisted of 306 men with localized PCa who have undergone radical prostatectomy. None of the 306 patients received preoperative radiation or androgen deprivation therapy. The second cohort included 9 patients diagnosed with neuroendocrine prostate cancer (NEPC) by biopsy. The 9 patients underwent either observation or surgery as initial treatment.

Morphology was validated by two pathologists (B.H. and X.L.). Detailed clinical and pathological profile were obtained from medical records and maintained on a secure relational database. This study was approved by Shandong University Medical Research Ethics Committee and informed consent was obtained from each patient.

### Immunohistochemistry (IHC)

IHC was performed as described previously [27]. The slides were incubated with antibodies at 4 °C for overnight. For negative controls, the antibodies were replaced with PBS. For assessment of intensity, each field was graded semi-quantitatively on tree-tier scale (0 = none staining, 1 = weak staining, 2 = moderate staining, 3 = strong staining). For analysis, we combined both negative and weakly BTF3 positive tumors into one group and compared it with moderately and strongly BTF3 positive PCa. Scoring was evaluated blindly by two independent observers (B.H and X.L.). Antibodies are described in Additional file 3: Table S1.

### Immunofluorescence

Immunofluorescence was performed as previously described [27]. For mouse-derived prostate tissue, deparaffinized and rehydrated slides were incubated with antibodies at 4 °C overnight, followed by Alexa Fluor-594 or − 488-conjugated secondary antibodies (Proteintech, Chicago, USA). Pretreated PCa cells were seeded on glass coverslips, fixed and then incubated with primary antibodies overnight at 4 °C, followed by secondary antibodies incubation. Antibodies used in this experiment are described in Additional file 3: Table S1.

### Cell lines, reagents and transfection

Human prostate cancer cell lines (VCaP, PC3, 22RV1, DU145 and LNCaP), human prostate epithelial cell line (RWPE) and 293 T(CRL-3216) were obtained from the American Type Culture Collection (Rockville, MD, USA) and cultured following the manufacturer’s recommendations. Detailed information of protocols for transient and stable transfection are available in Additional file 2: Materials and Methods.

### Tumor xenografts

Nude mice (nu/nu, male 3–4 weeks old) were subcutaneously injected with 2 × 10^6^ PC3 stable cells. The tumor growth was monitored and measured with calipers every 3 days. All animal experiments were conducted in strict accordance with the principles and procedures approved by the Shandong University Animal Care Committee.

### In vitro proliferation, migration and invasion assays

Cellular proliferation was measured by MTS (Promega, Madison, WI, USA), Cell-Light™ EdU DNA Cell Proliferation (EdU) (Ribobio, Guangzhou, China) and clonal assays. The transwell assays were used to measure the migration and invasion of PCa cells. These assays were performed as previously described [27].

### Clonal, clonogenic, and sphere-formation assays

Assays were performed as previously described [28]. The details of the procedures and analyses are described in Additional file 2: Materials and Methods.

### RNA extraction and quantitative real time PCR (RT-PCR)

Total RNA was extracted with Trizol reagents (Invitrogen) and mRNA levels were assayed by ReverTra Ace qPCR RT kit and SYBR Green PCR kit (Toyobo, Japan). GAPDH was used as an endogenous control. Sequences of primers used are given in Additional file 3: Table S1.

### Western blot

Western blot analysis was performed as previously described [29]. The membranes were incubated overnight with antibodies. Immunoreactivity was visualized using an enhanced chemiluminescence kit (Millipore, Darmstadt, Germany). Antibodies used in this study are described in Additional file 3: Table S1.

### Flow cytometry

Cells lines were trypsinized and stained with antibodies. All cells were analyzed in a CyAn ADP flow cytometer (Beckman Coulter) and processed with CytExpert software (Beckman Coulter). CD133 staining was performed with anti-CD133 (CD133/(AC133)-PE, human, #130–098-826).

### MRNA profiling and bioinformatics analysis

Microarray-based human gene expression profiling (KangCheng, Shanghai, China) was used to compare the mRNA expression profiles in control (Scr) and BTF3 stable knockdown (shBTF3) VCaP cells. Microarray data was deposited to the NCBI’s Gene Expression Omnibus (GEO) Repository and are now accessible through GEO series accession number GSE122749.

The The Cancer Genome Atlas (TCGA) and Memorial Sloan Kettering Cancer Center (MSKCC) datasets were downloaded from (http://gdac.broadinstitute.org/). Datasets of GSE35988, GSE68545, GSE68907, GSE6099, GSE19704, GSE77379, and GSE53902 were downloaded from GEO database (http://www.ncbi.nlm.nih.gov/geo). The expressed genes were subsequently analyzed for enrichment of biological themes using Gene Set Enrichment Analysis (GSEA) (http://software.broadinstitute.org/gsea/index.jsp). Signatures for stemness of PCa were derived from GSE19704, GSE77379 and GSE53902 (genes with *P* < 0.05, fold change> 2).

### Statistical analysis

Statistical analysis was carried out using GraphPad Prism 5 or SPSS 20.0 software, with *p* < 0.05 considered to be statistically significant. Refer to Additional file 2: Supplementary Materials and Methods for details.

## Results

### BTF3 is expressed in stem cell enriched population of prostatic tissues and PCa

**Fig. 1:**
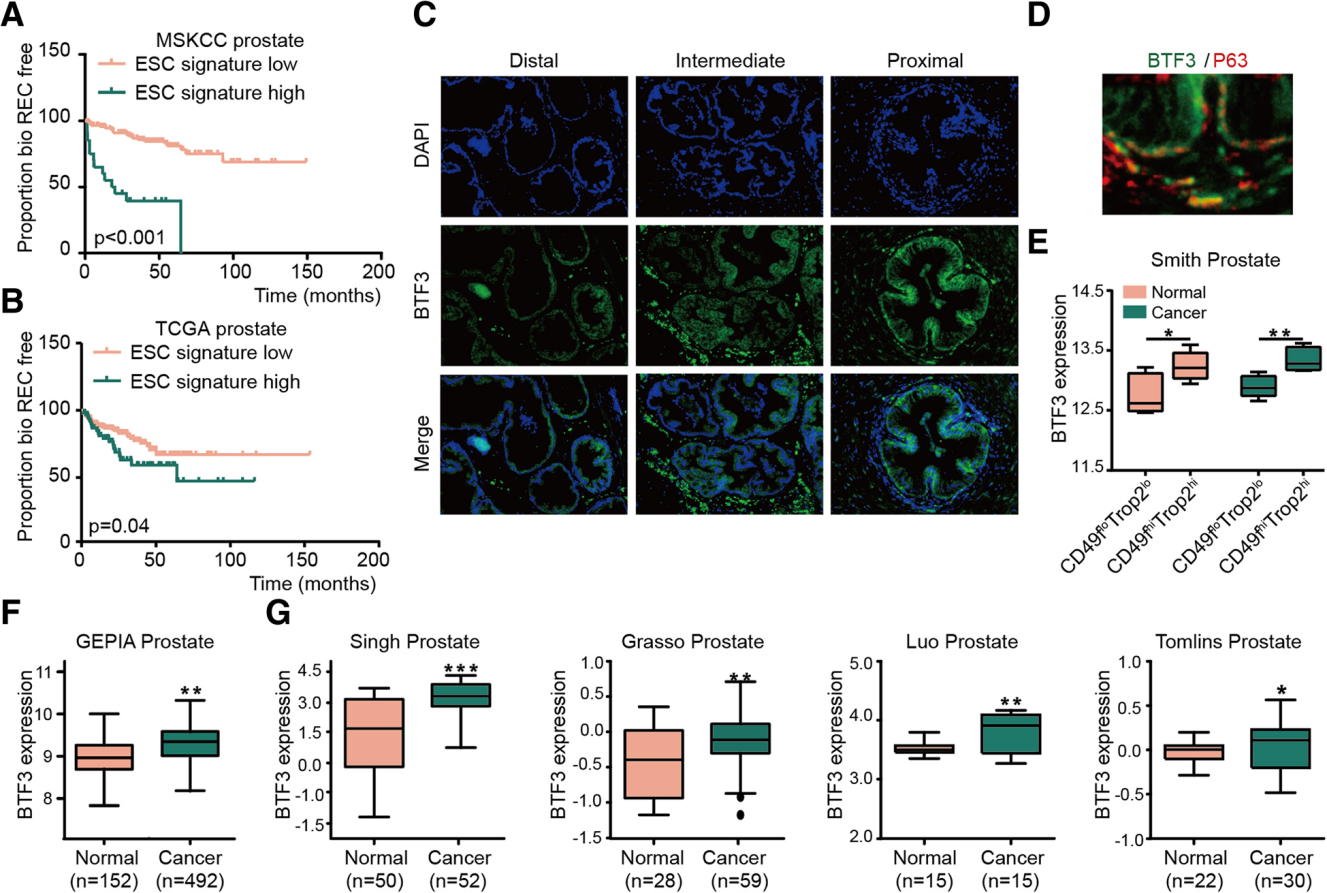
BTF3 expression is correlated with poor prognosis of prostate cancer (PCa) (**a**-**b**) Kaplan–Meier survival analysis of PCa cases from MSKCC prostate (**a**, p < 0.001, Log-rank test) and TCGA prostate (**b**, p = 0.04, Log-rank test) cohort according to high and low ESC signature expression. REC = recurrence. (**c**) Immunofluorescence analysis of BTF3 expression in proximal, intermediate and distal region of prostate from mouse. (**d**) Immunofluorescence analysis of BTF3 and P63 expression in proximal region of prostate from mouse. (**e**) Expression of BTF3 in CD49f^hi^Trop2^hi^ population compared with CD49f^lo^Trop2^lo^ population in Smith prostate cohort. CD49f^hi^Trop2^hi^ = CD49f high and Trop2 high; CD49f^lo^Trop2^lo^ = CD49f low and Trop2 low. (**f**-**g**) Expression of BTF3 in PCa tissues compared with normal prostate samples in GEPIA Prostate (**f**), Singh Prostate, Luo Prostate, Grasso Prostate and Tomlins Prostate. * *P* < 0.05, **P < 0.01, ***P < 0.001.

*BTF3* is one of the genes involved in ESC signature [6]. As shown in Fig. 1a-b, PCa patients with high expression of the ESC signature exhibited poor outcomes. We then analyzed the expression of BTF3 in PrSCs and PCa tissues. It has been documented that PrSCs with stem-like capabilities like self-renewal, multilineage differentiation and tissue regeneration are highly enriched in the proximal region of mouse prostate and mainly the basal compartment [30, 31]. As shown in Fig. 1c, cells in the proximal region of mouse prostate expressed higher levels of BTF3 and co-expressed the basal cell marker P63 (Fig. 1d). CD49f^hi^Trop2^hi^ marks novel prostate progenitor basal stem cell and aggressive PCa cells [32, 33]. CD49f^hi^Trop2^hi^ cells expressed higher *BTF3* levels as compared to the CD49f^lo^Trop2^lo^ population, either normal prostatic tissues or PCa (Fig. 1e). We next analyzed publicly available human PCa datasets for *BTF3* expression. After extensive research on the different datasets, including GEPIA prostate (Fig. 1f), Singh prostate, Grasso prostate, Luo prostate and Tomlins prostate (Fig. 1g), we determined that PCa expresses more *BTF3* than benign prostatic tissues. Taken together, these results suggest that BTF3 is correlated with stemness in prostatic tissues and upregulated in PCa.

### BTF3 is an activator of stem-like properties in PCa

**Fig. 2:**
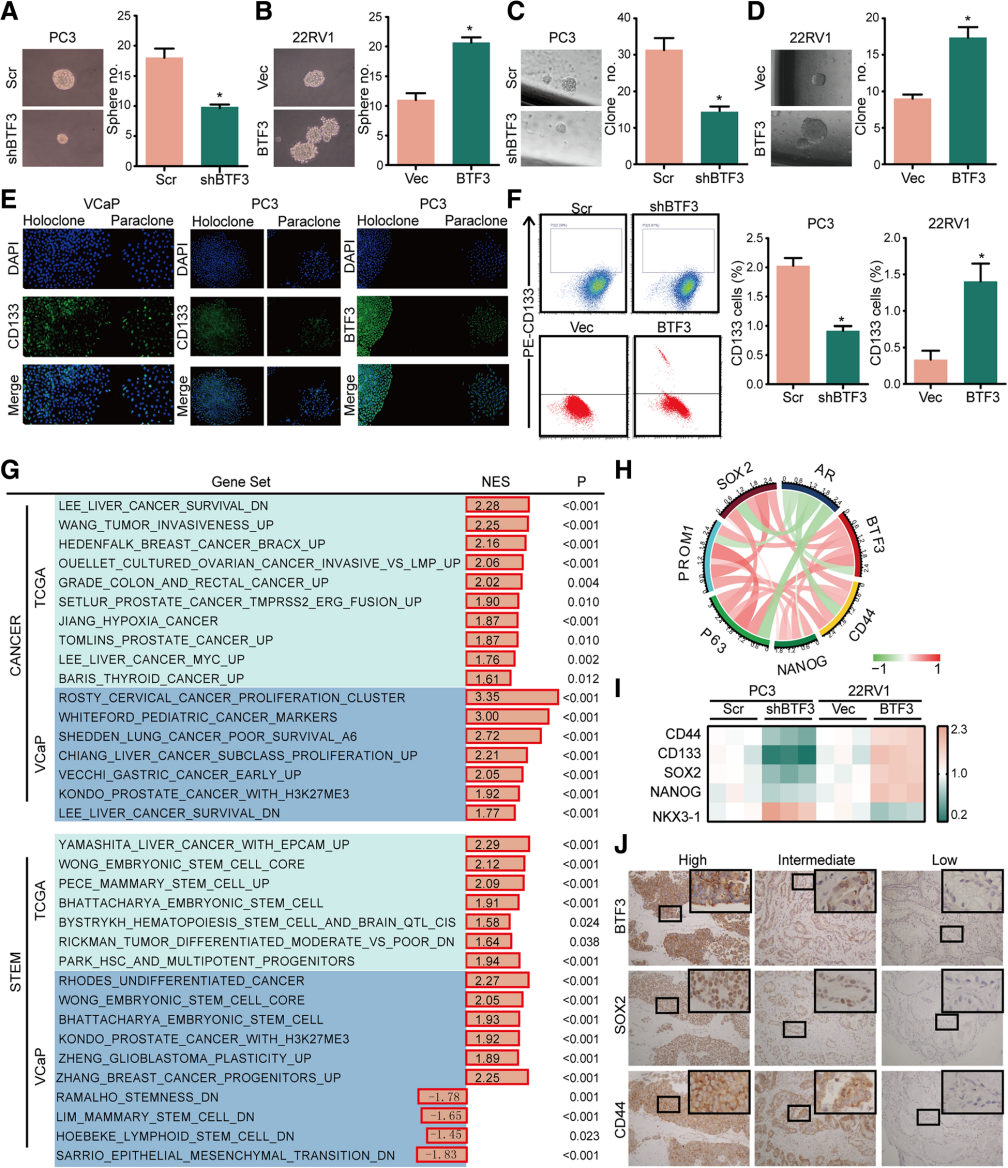
BTF3 expression is correlated with stem phenotype in PCa (**a**-**b**) Sphere formation assay of PC3 (**a**) and 22RV1 (**b**) cells. Left panel: Representative images of spheres. Right panel: Quantitative results of sphere formation assays from triplicate experiments. Scr/shBTF3: BTF3 was knockdown in PC3 cells by transfection of shRNA targeting BTF3 (shBTF3) or a negative control (Scr). Vector/BTF3: ectopic expression of BTF3 in 22RV1 cells by transfection of plasmid including BTF3 gene sequence (BTF3) or empty plasmid (Vector). (**c**-**d**) Clonogenic assays in PC3 (**c**) and 22RV1 (**d**) cells. Cells transfected as above were mixed with Matrigel and plated in 24-well plates and colonies counted in two weeks. Left panel: Representative images of clonogenic assay. Right panel: Quantitative results of clonogenic assay from triplicate experiments. (**e**) Expression of CD133 and BTF3 in holoclones and paraclones derived from VCaP and PC3 cells by immunofluorescence analysis. (**f**) Flow cytometry assay with CD133-PE antibody of indicated PCa cells. Left panel: FACS for CD133 indicated PCa cells. Right panel: Quantitative results of FACS from triplicate experiments. (**g**) Gene sets significantly enriched in high-BTF3 tumors grouped accordingly to the original annotation in the MSigDB Database C2 (http://software.broadinstitute.org/gsea/msigdb/index.jsp). Normalized enrichment score (NES) and *P*-value (P) are reported. TCGA-PRAD, the Cancer Genome Atlas Prostate Adenocarcinoma dataset (*N* = 493); VCaP, gene expression microarray assay of VCaP Scr/shBTF3 cells. (**h**) Circos plot displaying the interconnectivity among genes related to stemness phenotype and BTF3. The thickness and color of the ribbons correlate to the correlation of genes expression in Tomlins Prostate. (Additional file 4**: Table S2**) (**i**) The mRNA level of CSC markers, CD44, CD133, SOX2 and NANOG determined by real time PCR in indicated PCa cells. PC3 and 22RV1 cells transfected as above were subjected to real time PCR assay. (**j**) Representative IHC images of BTF3, SOX2 and CD44 expression of PCa tissue with different intensity in Qilu cohort. Each row represents one case. * *P* < 0.05, **P < 0.01, ***P < 0.001.

We examined BTF3 expression in the immortalized non-tumorigenic prostate epithelial cell line (RWPE) and a series of PCa cell lines (LNCaP, DU145, 22RV1, VCaP and PC3) (Additional file 1: Figure S1A) and performed gain- and loss-of-function analyses. VCaP and PC3 cells were chosen for BTF3 inhibition, while 22RV1, LNCaP and RWPE cells were chosen for BTF3 overexpression. (Additional file 1: Figure S1B). We analyzed the effects of BTF3 on self-renewal properties of PCa cells. As shown in Fig. 2a & Additional file 1: Figure S1C, BTF3 loss significantly impaired secondary sphere formation in PC3 cells, whereas forced expression of BTF3 increased prostasphere initiation and growth in 22RV1 cells (Fig. 2b). Clonogenic assay (in Matrigel) demonstrated that BTF3 inhibition displayed much fewer and smaller colonies, whereas BTF3 overexpression enhanced clonogenicity (Fig. 2c, d& Additional file 1: Figure S1D). Human PCa cell holoclones contain self-renewing stem cells whereas meroclones and paraclones containing more mature and differentiated cells [34, 35]. As shown in Fig. 2e, immunofluorescence analyses of clones derived from VCaP and PC3 cells showed that CD133+ cells enriched in holoclones and holoclones stained for BTF3 (Fig. 2e & Additional file 1: Figure S1E). Although not every PCa cell line expresses a significant amount of CD133, in cell lines of BTF3 inhibition resulted in a striking decrease of CD133+ subpopulation, and BTF3 overexpression led to increased percentage of CD133+ stem cell subpopulation (Fig. 2f& Additional file 1: Figure S1F).

In order to better understand the BTF3-induced phenotype change, we performed GSEA in TCGA dataset and VCaP Scr/shBTF3 array. Among the top scoring gene sets in high-BTF3 (*P* < 0.05, FDR < 0.1), we found several gene sets associated with cancer onset and progression (Fig. 2g). Interestingly, GSEA revealed the enrichment of gene sets functionally associated with ESCs and CSCs (Fig. 2g) including the ESC signature [6], and CSC signatures of PCa driven from GSE77376 and GSE19704 (Additional file 1: Figure S1G-H). In silico analysis revealed a positive correlation between *BTF3* expression and CSC markers, *PROM1*, *CD44*, *SOX2* as well as *NANOG*. These CSC markers, plus *BTF3*, showed positive correlation to the basal cell marker *P63*, and negative to the luminal cell marker *AR* (Fig. 2h).

Knockdown of the endogenously expressed BTF3 in PCa cells and PrSCs (derived from second sphere formation of PC3 cells) resulted in downregulation of CSC markers and upregulation of *NKX3–1* (Fig. 2i, Additional file 1: Figure S1I, S1J & S1K). In contrast, ectopic expression of BTF3 resulted in the opposite effect (Fig. 2i). To confirm the relationship between BTF3 expression and stem-like characteristics in PCa patients, we also analyzed the correlation of BTF3 expression and stemness markers (CD44 and SOX2) by IHC. Indeed, increased expression of BTF3 coordinated with increased stemness markers (Table 1, CD44, *p* = 0.042; SOX2, *p* = 0.037). Representative IHC images were shown in Fig. 2j. Taken together, BTF3 limits the amplitude of the stem cell program and modulates the equilibrium between CSCs and non-CSCs.

**Table 1:** Clinicopathological analysis of BTF3 expression in prostate cancer

Variables	BTF3 expression		Total	*P* Value
	Negative and weak	Moderate and strong		
Age (years)				0.279
< 65	16 (53.3)	14 (46.7)	30	
> =65	114 (63.7)	65 (36.3)	179	
Pre PSA (ng/ml)				0.727
< 4	9 (56.3)	7 (43.7)	16	
4–10	15 (68.2)	7 (31.8)	22	
> =10	94 (65.3)	50 (34.7)	144	
Gleason Score				0.006
< 7	31 (83.8)	6 (16.2)	37	
7	75 (72.8)	28 (27.2)	103	
> 7	96 (59.6)	65 (40.4)	161	
Stage				0.02
T1-T2	98 (65.8)	51 (34.2)	149	
T3-T4	22 (46.8)	25 (53.2)	47	
Metastasis				0.005
Negative	94 (67.1)	46 (32.9)	140	
Positive	24 (45.3)	29 (54.7)	53	
Ki67				0.062
< 10%	128 (65.6)	67 (34.4)	195	
> 10%	10 (45.5)	12 (54.5)	22	
Biochemical recurrence				0.251
Negative	17 (65.4)	9 (34.6)	26	
Positive	42 (52.5)	38 (47.5)	80	
Clinical recurrence				< 0.001
Negative	84 (72.4)	32 (27.6)	116	
Positive	33 (46.5)	38 (53.5)	71	
CD44				0.042
Negative	32 (91.4)	3 (8.6)	35	
Positive	18 (69.2)	8 (30.8)	26	
SOX2				0.037
Negative	39 (79.6)	10 (20.4)	49	
Positive	6 (50)	6 (50)	12	

### BTF3 regulates growth and invasion though modeling stem-like characteristics of PCa cells

**Fig. 3:**
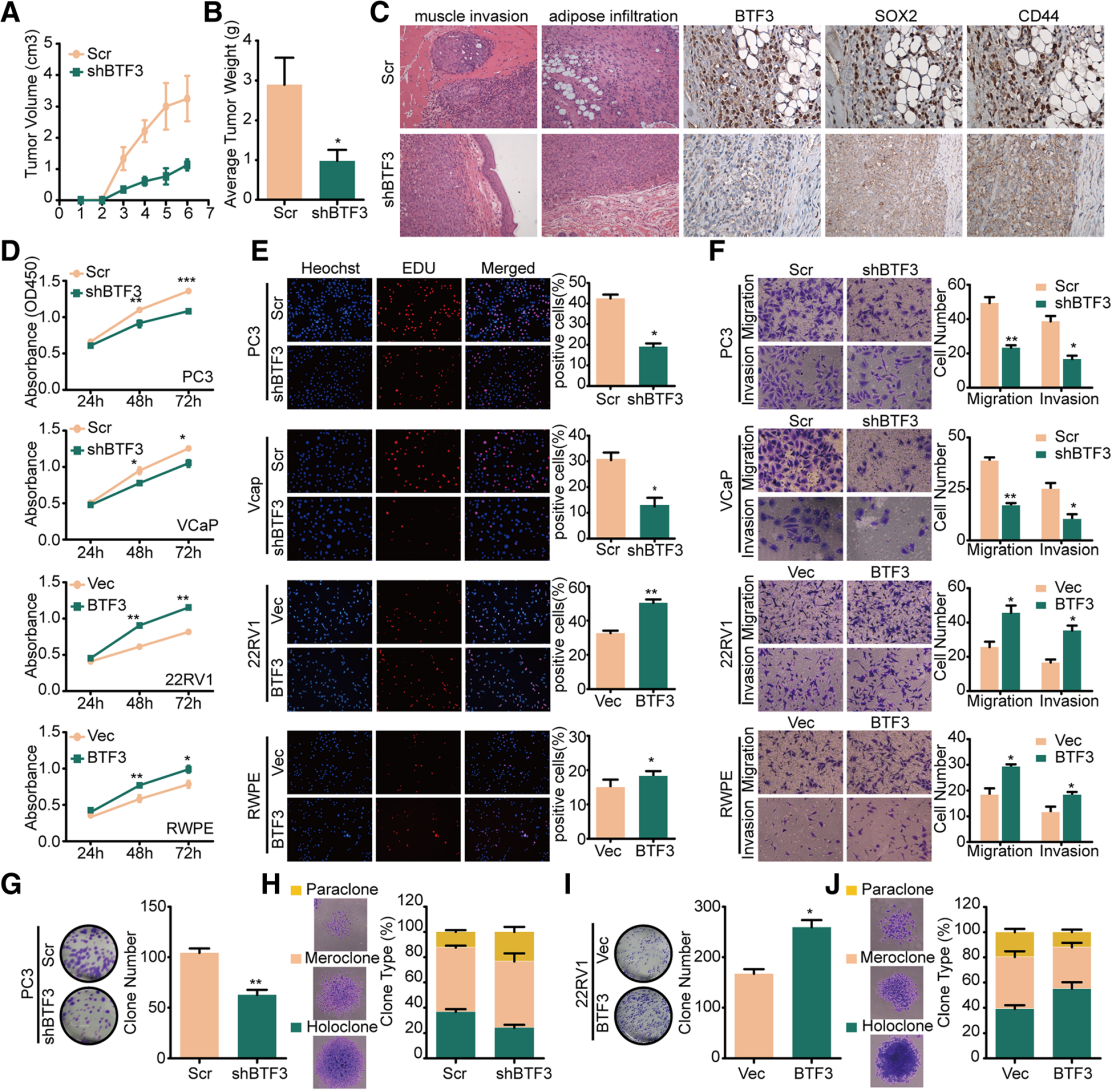
BTF3 promotes proliferation and invasion of PCa cells though modeling stem traits of PCa (**a**-**b**) Effect of BTF3 on turmorigenesis in vivo evaluated with xenografts model. PC3 cells with stable expression of Scr/shBTF3 subcutaneously injected into nude mice. Tumors were measured every 3 days using a vernier calliper, and the volume was calculated according to the formula: 1/6 x length x width^2^. Growth curves of tumors (**a**), and the average weight of tumor mass in each group (**b**) were shown. (**c**) Representative H&E and IHC images of xenograft tumor derived from PC3 Scr/shBTF3 cells. HE and IHC stain were performed to each tumor at harvest time. (**d**) Cell viability as assessed by MTS assay of indicated PCa cell lines. (**e**) EdU assays of indicated PCa cell lines transfected with Scr/shBTF3 or Vec/BTF3. Left panel: Representative images of EdU assay. Right panel: Quantitative results of EdU assays from triplicate experiments. (**f**) The effect of BTF3 on cell migration/invasion evaluated cells by transwell migration and matrigel invasion assays. PCa cell lines were transfected with Scr/shBTF3 or Vec/BTF3 as indicated. Left panel: Representative images of cell migration and invasion. Right panel: Quantitative results of migration and invasion assays from triplicate experiments. In each experiment, cells were counted in five random fields of each filter under a microscope using a 40 × magnification. (**g**-**j**) Clonal assay of PC3(G-H) and 22RV1 (I-J) cells with BTF3 ablation or expression. Cells were cultured for two weeks followed by staining with Giemsa and photography. Total number of all clones (**g** &**i**) and summary of three types of clones (**h** &**j**) presented as representative images (left) and quantification (right). * *P* < 0.05, **P < 0.01, ***P < 0.001.

The ability of cancer cells to grow or to metastasize has been linked to the presence of CSCs [36]. To test whether this change in the stem cell traits induced by BTF3 has consequences for the ability of PCa cells to proliferate and invade, we investigated the role of BTF3 on tumor growth using a xenograft model. As shown in Fig. 3a, b & Additional file 1: Figure S2A, the tumors derived from PC3 cells with BTF3 depletion grew less rapidly than those derived from the control group. At the endpoint, the average tumor volume and weight were significantly reduced in shBTF3 group (Fig. 3c). The Scr group showed evident infiltration into muscle and adipose tissues, and formed tumor thrombus in venous vessels. The shBTF3 group generally showed expansive growth with continuous pseudocapsule (Fig. 3c& Additional file 1: Figure S2B). Moreover, IHC analysis showed decreased SOX2 and CD44 expression in shBTF3 group (Fig. 3c). To extend the in vivo observations, we investigated the role of BTF3 in PCa cells in vitro. We found that BTF3 down-regulation significantly decreased cell proliferation whereas BTF3 overexpression promoted cell growth, as measured by MTS assay (Fig. 3d& Additional file 1: Figure S2C) and EdU assay (Fig. 3e). Knockdown of BTF3 significantly suppressed migration and invasion while overexpressing BTF3 had the opposite effect in PCa cells (Fig. 3f).

Colony formation assay further confirmed that BTF3 inhibition in PCa cells impaired clonogenic growth as compared to the controls (Fig. 3g, Additional file 1: Figure S2D & Figure S2E). Importantly, BTF3 inhibition led to a decrease in the proportion of holoclones (Fig. 3h & Additional file 1: Figure S2F). Ectopic expression of BTF3 promoted clonogenic growth and holoclones formation (Fig. 3i & j).

Together, these experiments show that BTF3 inhibition mediated suppression of prostate stem-like phenotype which resulted in a block of tumor growth and progression, which may be utilized as a treatment strategy in patients.

### BMI1 is a direct target of BTF3 in PCa

**Fig. 4:**
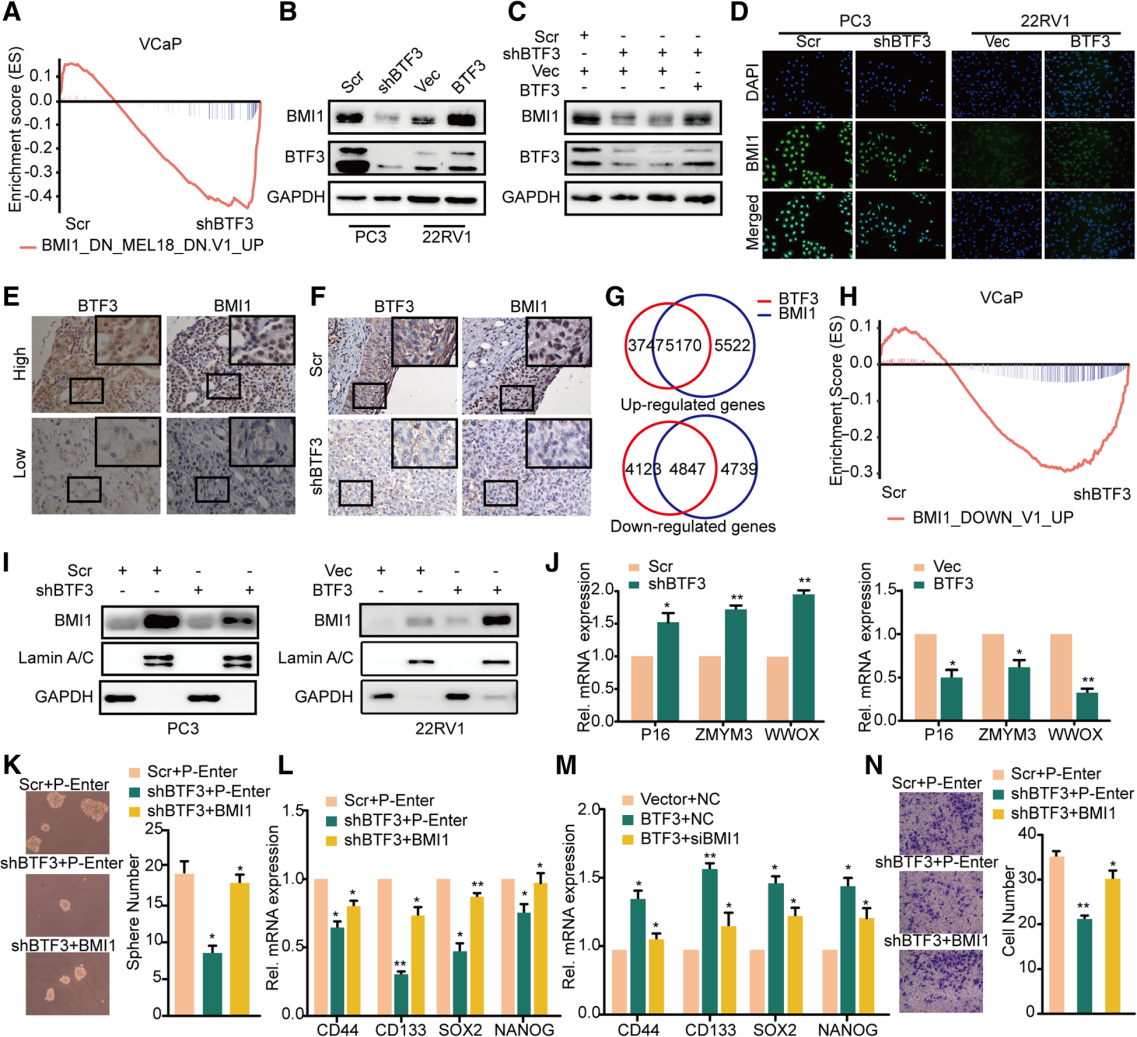
BMI1 is a BTF3 target gene (**a**) BMI1 downregulated gene signatures (MSigDB Database C6) were enriched for upregulation upon BTF3 knockdown. GSEA was carried out to examine the expression of a set of BTF3-repressed genes in a microarray dataset that profiled VCaP cells with control or BTF3 knockdown. ES = -0.44, *P* = 0.001, FDR *q* = 0.02. (**b**-**c**) Western blot analysis of BTF3 and BMI1 expression in PCa cells with BTF3 knockdown or overexpression. (**d**) Immunofluorescence staining analysis of BTF3 and BMI1 expression in PCa cells. (E) Representative images of HE and IHC staining of BTF3 and BMI1 in PCa patients from Qilu cohort. (**f**) Representative IHC images of BTF3 and BMI1 expression in xenograft tumors derived from PC3 cells. (**g**) Overlapped genes regulation by BTF3 and BMI1 through mRNA profiling analysis. (**h**) GSEA of BMI1 signatures (genes upregulated upon BMI1 inhibition) in a microarray dataset that profiled VCaP cells with control or BTF3 knockdown. ES = -0.3, *P* = 0.022, FDR *q* = 0.022. (**i**) Cytoplasmic and nuclear BMI1 protein levels were analyzed in PC3 and 22RV1 cells after shBTF3 or BTF3 overexpression. GAPDH and LaminA/C were used as cytoplasmic and nuclear protein-loading controls, accordingly. (**j**) The mRNA levels of P16, ZMYM3 and WWOX in PCa cells were detected by qRT-PCR in the presence of Scr or shBTF3 (left), and Vector or BTF3 (right). Values represent mean ± SD of technical duplicates from a representative experiment. (**k**) Sphere formation assay of PC3 cells. PC3 Scr/shBTF3 cells were transiently transfected with empty plasmid (P-Enter) or BMI1 expression plasmid (BMI1). Left panel: Representative images of spheres. Right panel: Quantitative results of sphere formation assays from triplicate experiments. (**l**-**m**) The mRNA level of CSC markers, CD44, CD133, SOX2 and NANOG determined by real time PCR in PC3(**j**) and 22RV1 (**k**) cells. (**n**) Effect of BMI1 in Transwell migration of BTF3. PC3 Scr/shBTF3 cells with were transiently transfected with empty plasmid (P-Enter) or BMI1 expression plasmid (BMI1) as indicated. Cells were subjected to transwell migration assay. * P < 0.05, **P < 0.01, ***P < 0.001.

GSEA suggested that several oncogenic signatures including signatures associated with MYC, EZH2, BMI1 etc., were enriched in BTF3 regulation. However, only BMI1 was suggested to bind to BTF3 by mass spectrum. By integrating the mass spectrum and GSEA results of gene expression array, we presumed that BMI1 was a downstream target of BTF3. GSEA indicated that BMI1 downregulated gene sets were significantly enriched in VCaP shBTF3 cells (Fig. 4a). Western blot assay further revealed that BMI1 protein expression was significantly inhibited in PCa cells with BTF3 knockdown but increased with BTF3 overexpression (Fig. 4b& Additional file 1: Figure S3A). As expected, ectopic expression of BTF3 in PC3 shBTF3 cells restored BMI1 expression (Fig. 4c). Concordantly, the regulation of BTF3 on BMI1 expression was verified by immunofluorescence (Fig. 4d). We next examined whether BTF3 expression is relevant to BMI1 status in vivo. Interestingly, a positive correlation of BTF3 and BMI1 expression was observed in both clinical specimens (Fig. 4e) and xenograft tumors (Fig. 4f) at the protein level. However, no remarkable correlation of BTF3 and BMI1 was identified at mRNA level (Additional file 1: Figure S3B & Figure S3C). We next investigated BMI1 function under BTF3 manipulation. By mRNA profiling analysis, BTF3 and BMI1 showed overlap of gene regulation (Fig. 4g). Genes negatively related to BMI1 were enriched in the BTF3 inhibited sample (Fig. 4h). Importantly, nuclear translocation of BMI1 could be significantly inhibited with BTF3 knockdown and was increased with BTF3 expression (Fig. 4i). The expression of well documented target genes of BMI1 (*P16*, *ZMYM3* and *WWOX*), was also affected by BTF3 manipulation. Thereby we confirmed that BMI1 is a target of BTF3 (Fig. 4j).

As shown in Fig. 4k, BTF3 loss alone significantly impaired sphere formation, while BMI1 expression partially restored the sphere formation ability. Further analysis showed that BTF3 loss significantly decreased the expression of CSC markers (*CD44*, *CD133*, *SOX2* and *NANOG*), and BTF3 expression increased the expression level. More importantly, the changes in CSC markers expression were partially reversed by BMI1 expression or knockdown (Fig. 4l-m). Similar results were found by Transwell and MTS assays (Fig. 4n & Additional file 1: Figure S3D). Therefore, BTF3 induced stemness traits though BMI1 regulation in PCa cells.

### BTF3 stabilizes BMI1 proteins

**Fig. 5:**
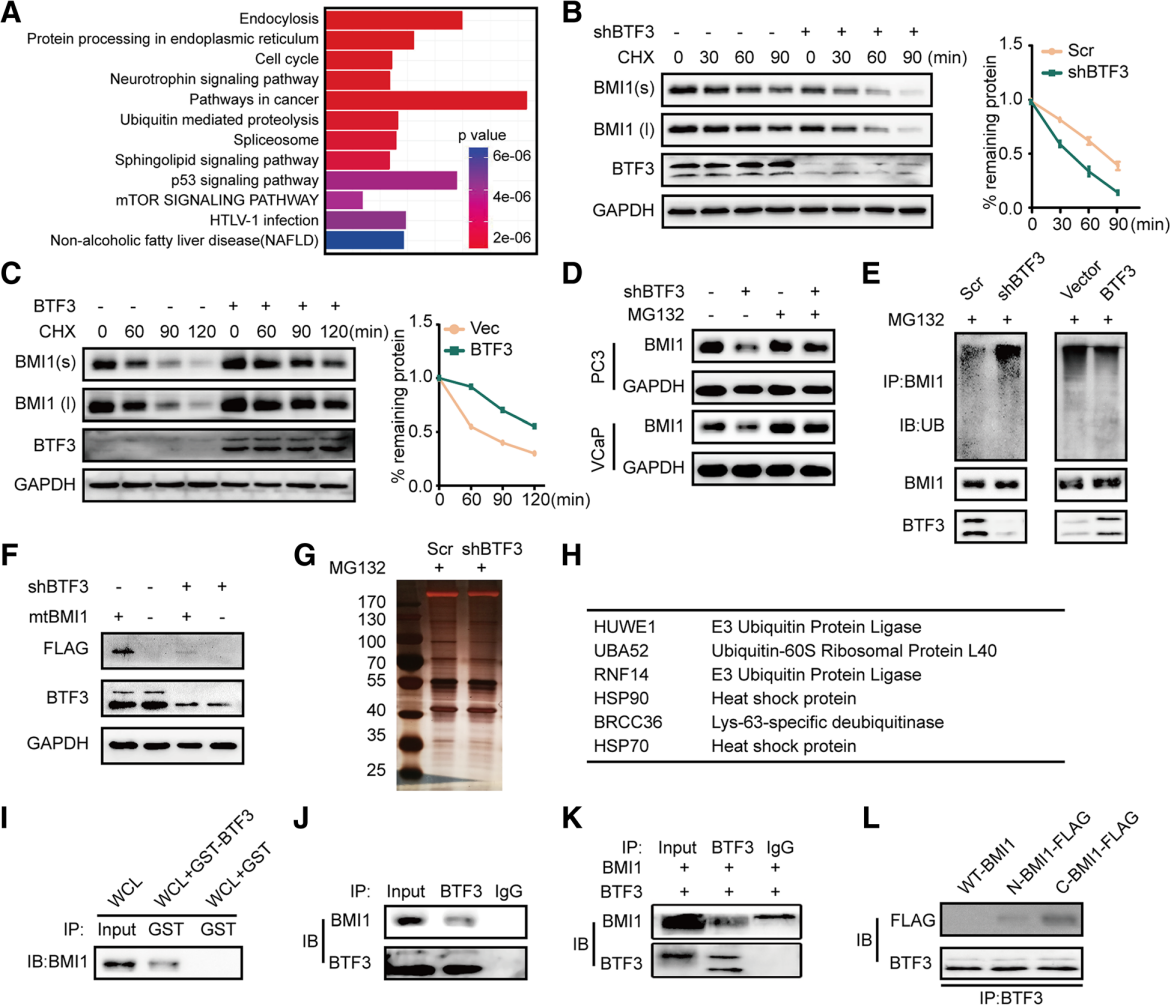
BTF3 interacts and stabilizes BMI1 protein (**a**) Kyoto Encyclopedia of Genes and Genomes (KEGG) terms of genes regulated by BTF3. (**b**-**c**) BMI1 protein levels were determined after incubation with cyclohexamide (CHX, 10 μg/mL) for the indicated time periods in PC3 Scr/shBTF3 (**b**) or 22RV1 Vec/BTF3 (**c**) cells. Left panel: Representative images of western blot assay. Right panel: the densitometric quantification of BMI1 normalized to GAPDH was plotted against various time points to determine its half life. BMI1(s): BMI1 with short exposure; BMI1(**l**): BMI1 with long time exposure. (**d**) PC3 and VCaP cells with Negative control (Scr) or BTF3 knockdown (shBTF3) were treated with control or 10 mM MG132 for 6 h and subjected to western blot analysis. (**e**) Effects of BTF3 on BMI1 ubiquitination. PC3 Scr/shBTF3 and 22RV1 Vec/BTF3 cells were incubated with MG132 (20 μM) for 6 h. Cell lysates were immunoprecipitated with BMI1 antibody and then immunoblotted with ubiquitin antibody. BTF3 and BMI1 in whole cell lysates (input) were shown. (**f**) Expression analysis of BMI1 with mutant β-TrCP binding site (mtBMI1) by western blot assay. (**g**) Identification of interacting proteins with BMI1 by co-IP and mass spectrometry. PC3 Scr/shBTF3 cells were incubated with MG132 (20 μM) for 6 h. Cell lysates were immunoprecipitated with BMI1 antibody and subjected to SDS–PAGE. (**h**) Possible proteins involved in regulation of BMI1 by BTF3 according to mass spectrometry. (**i**) Western blot analysis of GST-pull down assays. Whole-cell lysates from HEK293T cells were incubated with GST or GST-BTF3 fusion proteins coupled sepharose beads. After washing to remove unbound proteins, they were subjected to SDS–PAGE and western blot with antibodies against GST. (**j**-**k**) Co-immunoprecipitation assays of BTF3 and BMI1 in PC3 (**g**) and 293 T (**h**) cells. 293 T cells were transfected with plasmid for BMI1 and BTF3 expression. Cell lysates were immunoprecipitated with IgG control antibody or BTF3 antibody and then immunoblotted with BMI1 antibody. (**l**) Co-immunoprecipitation assays of BTF3 and truncate BMI1 in 293 T cells. 293 T cells were transfected with WT-BMI1, N-BMI1-FLAG or C-BMI1-FLAG plasmids and then subjected to co-immunoprecipitation assays with BTF3 antibody and then immunoblotted with FLAG antibody. * P < 0.05, **P < 0.01, ***P < 0.001.

As shown in Fig. 5a, GSEA revealed that genes regulated by BTF3 enriched in the ubiquitination pathway. To test whether BTF3 modulates BMI1 expression through ubiquitin-mediated degradation, we treated PCa cells with CHX, a protein biosynthesis inhibitor. Western blot analysis showed that BTF3 loss remarkably accelerated the degradation of BMI1 protein (Fig. 5b). By contrast, BTF3 overexpression in 22RV1 suppressed the degradation of BMI1 protein (Fig. 5c). Furthermore, immunoblotting analysis showed that BTF3 loss failed to decrease the protein level of BMI1 in the presence of proteasome inhibitor MG132 (Fig. 5d). Immunoprecipitation assays showed that, with MG132, BTF3 knockdown increased levels of ubiquitinated BMI1 whereas BTF3 overexpression decreased the levels (Fig. 5e). These findings suggest that BTF3-facilitated stabilization of BMI1 protein depends on proteasome-mediated degradation.

BMI1 was documented to be ubiquitinated by β-TrCP [37]. To investigate whether β-TrCP was involved in the regulation of BMI1 by BTF3, we constructed BMI1 expression plasmids with mutant β-TrCP degron site. Our results showed the expression of mutant BMI1 was downregulated by BTF3 loss (Fig. 5f). Therefore, β-TrCP was not involved in the regulation of BMI1 by BTF3. Next, we performed mass spectrometry of BMI1 binding protein under BTF3 alteration (Fig. 5g). The mass spectrometry results suggested several proteins associated with protein stability may involve in the regulation of BMI1 by BTF3 (Fig. 5h).

### BTF3 physically interacts with BMI1

GST pull-down experiment suggested a directly interaction between BTF3 and BMI1 protein (Fig. 5i). The interaction was further confirmed by co-immunoprecipitation (Co-IP) in VCaP cells endogenously expressing BTF3 and BMI1 (Fig. 5j), and HEK 293 T cells ectopic expressing BTF3 and BMI1 protein (Fig. 5k). To identify the domain that BTF3 binds to BMI1, we generated truncated mutants of BMI1 according to the protein domain structure (Additional file 1: Figure S4B) and performed Co-IP assays (Fig. 5l). Results showed that BTF3 interacted with the C-terminal part of BMI1 (C-BMI1, aa160–326) while the N-terminal part (N-BMI1, aa1–170) did not interact with BTF3. Taken together, these results suggest that BTF3 physically interacts with BMI1.

### High expression of BTF3 predicates aggressive PCa and poor outcomes

**Fig. 6:**
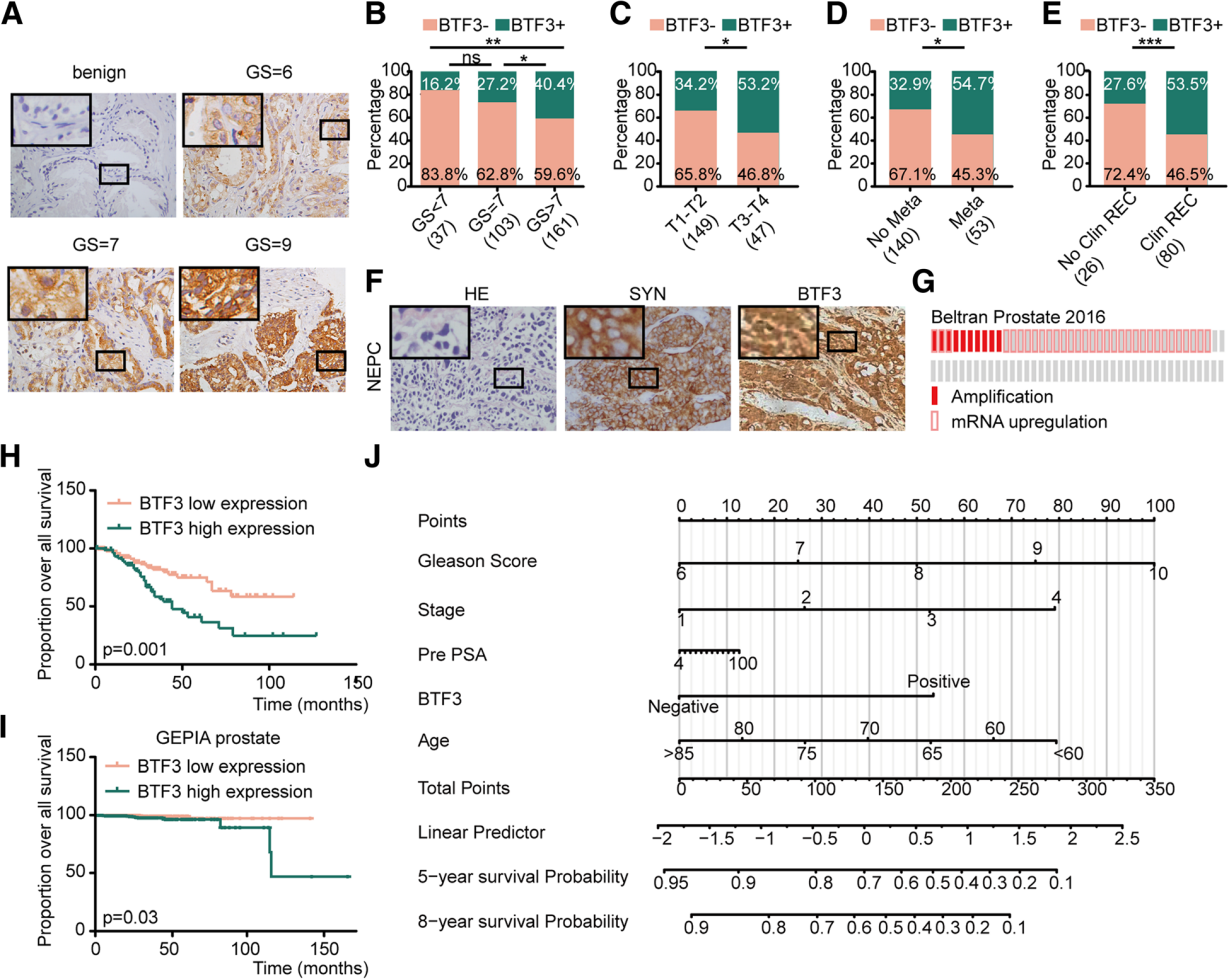
BTF3 overexpression predicts poor outcome of PCa (**a**) Representative IHC images of BTF3 expression of benign prostate tissue and PCa tissue with different Gleason score in Qilu cohort. GS = Gleason score. (**b**-**e**) The percentage of BTF3 expression distributed in PCa cases with different Gleason score (**g**), stage (**h**), metastasis(**i**) and clinical recurrence(**i**). REC = recurrence. (**f**) Representative HE and IHC images of NEPC tissue showing SYN and BTF3 expression. (**g**) Oncoprint of amplification and mRNA upregulation of BTF3 gene in Beltran Prostate cohort according to the cBioPortal database. (**h**) Kaplan–Meier survival analysis of PCa cases from Qilu cohort stratified by high and low BTF3 expression levels (*p* = 0.006, Log-rank test). (**i**) Kaplan–Meier survival analysis of PCa cases from GEPIA cohort according to high and low BTF3 expression levels (*p* = 0.03, Log-rank test). (**j**) Nomograms based on Gleason score, stage, Pre PSA (4–10–20-30-40-50-60-70-80-90-100 ng/ml), BTF3 expression and Age for predicting survival probability of PCa patients.

We investigated a cohort of 306 Chinese PCa cases (Qilu cohort) from our hospital by IHC assay. As shown in Table 1, BTF3 overexpression in PCa was correlated with higher Gleason score (Fig. 6a, b, *p* = 0.006), clinical stage (Fig. 6c, *p* = 0.02) and metastasis (Fig. 6d, *p* = 0.005). However, there was no association between BTF3 expression and age or pre-treatment prostate specific antigen (Pre PSA). We next examined the relationship between the clinical outcomes of PCa patients and BTF3 expression. BTF3 overexpression in PCa was correlated with clinical recurrence (Fig. 6e, *p* < 0.001). However, there was no association between BTF3 expression and biochemical recurrence. Significantly, overexpression of BTF3 was identified in 66.7% (6/9) of PCa patients with small cell morphology (Fig. 6f). We also discovered that up to 48% of patients in Beltran Prostate cohort (composed of NEPC and castration resistant PCa) harbor BTF3 overexpression or amplification according to the cBioPortal database (Fig. 6g). Of note, patients with higher BTF3 expression exhibited worse overall survival in our PCa cohort (Fig. 6h) and GEPIA PCa cohort (Fig. 6i). In subgroup analysis of overall survival, higher expression of BTF3 suggested elevated hazad ratio (HR) in patients with high-risk PCa (Additional file 1: Figure S5A). Similar pattern was found in subgroup analysis of BTF3 on the risk of clinical recurrence (Additional file 1: Figure S5B). Moreover, univariate and multivariate analysis showed that BTF3 expression was an independent risk factor for clinical recurrence (Table 2, RR = 2.455, 95% CI: 1.19–5.065).

**Table 2:** Univariate and multivariate analysis for clinical recurrence in Qilu cohort (Cox proportional hazards regression model)

Variable	Univariate analysis			Multivariate analysis		
	P	RR	95%CI	P	RR	95%CI
BTF3	0.000	3.023	1.628–5.614	0.015	2.455	1.19–5.065
Age	0.057	0.474	0.219–1.023			
Pre PSA	0.886	0.967	0.615–1.521			
Gleason Score	0.000	2.55	1.624–4.003	0.014	2.019	1.154–3.534
Stage	0.000	4.493	2.246–8.99	0.14	1.952	0.802–4.749
Metastasis	0.000	10.396	5.021–21.522	0.000	5.736	2.511–13.102

To provide the clinician with a quantitative method to predict a patient’s probability of PCa survival, we constructed nomograms that integrated clinicopathological factors with BTF3 expression. The prognostic power of nomograms was assessed using C-Index (Additional file 1: Figure S6A). The nomogram including Stage, Pre PSA, Gleason Score, Age and BTF3 expression was chosen for further analysis (Fig. 6j). The calibration curve of nomogram was shown in Additional file 1: Figure S6B. The HR and area under ROC curve (AUC) were also calculated in subgroups of PCa to validate prognostic power of the nomogram (Additional file 1: Figure S6C). These results suggested that BTF3 expression was highly predictive of PCa prognosis.

## Discussion

Human cancers are heterogeneous containing CSCs that possess high capacities for tumor propagation and metastasis [38, 39]. CSCs populations have been identified to contribute to metastasis or castration, the two main handcuff of PCa treatment. In the current study, we discovered that BTF3 functions as a key regulator of PCa stemness traits mainly via stabilization of BMI1.

The unique ability of ESCs and ASCs to self-renew and give rise to multiple cell lineages have formed the basic definitions of “stemness” [6]. ESCs, ASCs and CSCs share similar transcriptional programs and also district from each other [40]. Genes involved in ESC or ASC signatures were found to be induced in many aggressive human epithelial cancers and links poor outcomes [6, 40]. Studies on genes associated with ESCs and ACSs in PCa progression help to define CSC characteristics and understand molecular mechanisms in PCa progression. The NAC family is a ribosome-associated chaperone that is important for protein homeostasis. NAC binds to unfolded, folded, or intrinsically disordered proteins to prevent aggregation. Recent studies revealed that NAC proteins have other functions. αNAC, was reported to act as a co-activator through interaction with phosphorylated c-JUN in osteoblasts [41]. αNAC deficiency results in impaired hematopoietic stem/progenitor cells maintenance [42] and erythroid-cell differentiation [43]. The other NAC protein, BTF3, interacts with Class II (B) general transcription factor and ERα to model transcriptional activity [44, 45]. BTF3 promotes the proliferation, survival, and migration of ER + breast cancer cells by modulating ESR1 expression and ERα-dependent transcription [26]. Wong et.al reported that BTF3 is associated with ESC signature [6]. The exact function and mechanisms of BTF3 in stem-maintenance and differentiation of ECSs still require further investigation. In this article, we reported that BTF3 is associated with PCa stem-like phenotype. Loss of BTF3 lead to attenuation of stemness, embodied in less sphere and holoclone formation, and decreased CSC markers. Furthermore, evidences from multiple experiments showed that BTF3 promotes proliferation and migration capacity though modulation of stem-like traits of PCa cells.

One of the key findings of the current study is the molecular mechanism underlying BTF3-promotion stemness traits of PCa. We are the first to demonstrated that BTF3 is a positive regulator of BMI1. BMI1 is a critical key regulator that controls the pluripotency of ESCs and early embryonic development [3, 11, 13, 14] . In an attempt to address how BTF3 regulates BMI1 expression, we focused on ubiquitination based mechanism. Mechanistic investigation indicates that BTF3 physically interacts with and inhibits proteasomal degradation of BMI1. The stabilized BMI1 complex then regulates its target genes to promote malignant behaviors and stemness of cancer cells. Intriguingly, the data clearly showed that silencing BMI1 by siRNA could reverse BTF3-induced stemness in PCa cells. These finding extend the previously known functions of NAC proteins.

The ubiquitin proteasome system is one of the important processes that ensure the integrity of proteostasis which as fundamental regulators of stemness [46, 47]. Interestingly, BTF3 as member of NAC family participates as key regulator of proteostasis [48]. Ubiquitination and degradation mediated by proteasome system play important roles in expression and activity of target proteins, including pluripotency factors. For example, OCT4 and SOX2 are ubiquitinated by the E3 ligase WWP2 during differentiation [49, 50]. MYC is degraded by E3 ligase Fbxw7 during ESC differentiation [47]. β-TrCP is also reported as a E3 ubiquitin ligase in the degradation of BMI1 [37]. BMI1 is a short-lived protein because it is actively degraded by the ubiquitin-proteasome pathway [37]. The mechanisms of BMI1 degradation besides β-TrCP interaction still requires further investigation. Interaction between BTF3 and BMI1 stabilized BMI1 from degradation and reinforced its function in self-renewal of PCa.

Although more and more PSA-screened PCa patients have been identified in Western countries, there is limited data regarding the clinical phenotype or natural history of PCa. Of note, our cohort included a large subset of patients with high grade PCa. This is different from most Western patients who were found to have PCa due to PSA screening. In this study, we developed a prognostic tool based on clinicopathological factors and BTF3 expression to improve the prediction of overall survival for patients with PCa. Our results showed that this tool successfully assisted risk stratification and predicts survival probability. This study is limited because it is retrospective and has limited generalizability as distribution of clinical characteristics may be different in other areas. However, our results merit further validation in other multicenter clinical trials.

## Conclusion

In conclusion, we showed that BTF3 activates stem-like phenotype of PCa and positively regulates BMI1 expression. Our findings reveal an intriguing role of BTF3 in modulating the tumorigenesis and progression of PCa cells.

## Additional files

Additional file 1: Supplementary Figures and Legends. **Figure S1.** BTF3 overexpressed in PCa. **Figure S2.** BTF3 functioned an oncogene in PCa. **Figure S3.** BTF3 targets BMI1 for stemness modeling of PCa cells. **Figure S4. Figure S5. Figure S6.** (PDF 1295 kb)

Additional file 2: Supplementary Materials and Methods. Cell lines and regents. Plasmid constructs and transfection. Clonal, clonogenic, and sphere-formation assays. Statistical analysis. (DOCX 17 kb)

Additional file 3: **Table S1.** xls Primers, siRNA and antibodies used in this study. (XLS 27 kb)

Additional file 4: **Table S2.** xls Correlation of BTF3 expression and CSC markers. (XLS 26 kb)

## Abbreviations

ASC: Adult tissue stem cell
BMI1: BMI1 proto-oncogene
BTF3: Basic transcription factor 3
CSC: Cancer stem cell
ESC: Embryonic stem cell
NAC: Nascent- polypeptide associated complex
NEPC: Neuroendocrine Prostate Cancer
PCa: Prostate cancer
Pre PSA: Pre-treatment prostate specific antigen
PrSC: Prostate stem cell
