# Supplementary material for: BTF3 sustains cancer stem-like phenotype of prostate cancer via stabilization of BMI1
Source: J Exp Clin Cancer Res. 2019 May 28;38:227. doi: 10.1186/s13046-019-1222-z (PMC6540453; doi:10.1186/s13046-019-1222-z)
Supplement: Supplementary file 1 — Supplementary Figures and Legends. Figure S1. BTF3 overexpressed in PCa. Figure S2. BTF3 functioned an oncogene in PCa. Figure S3. BTF3 targets BMI1 for stemness modeling of PCa cells. Figure S4. Figure S5. Figure S6. (PDF 1295 kb) [file 13046_2019_1222_MOESM1_ESM.pdf]

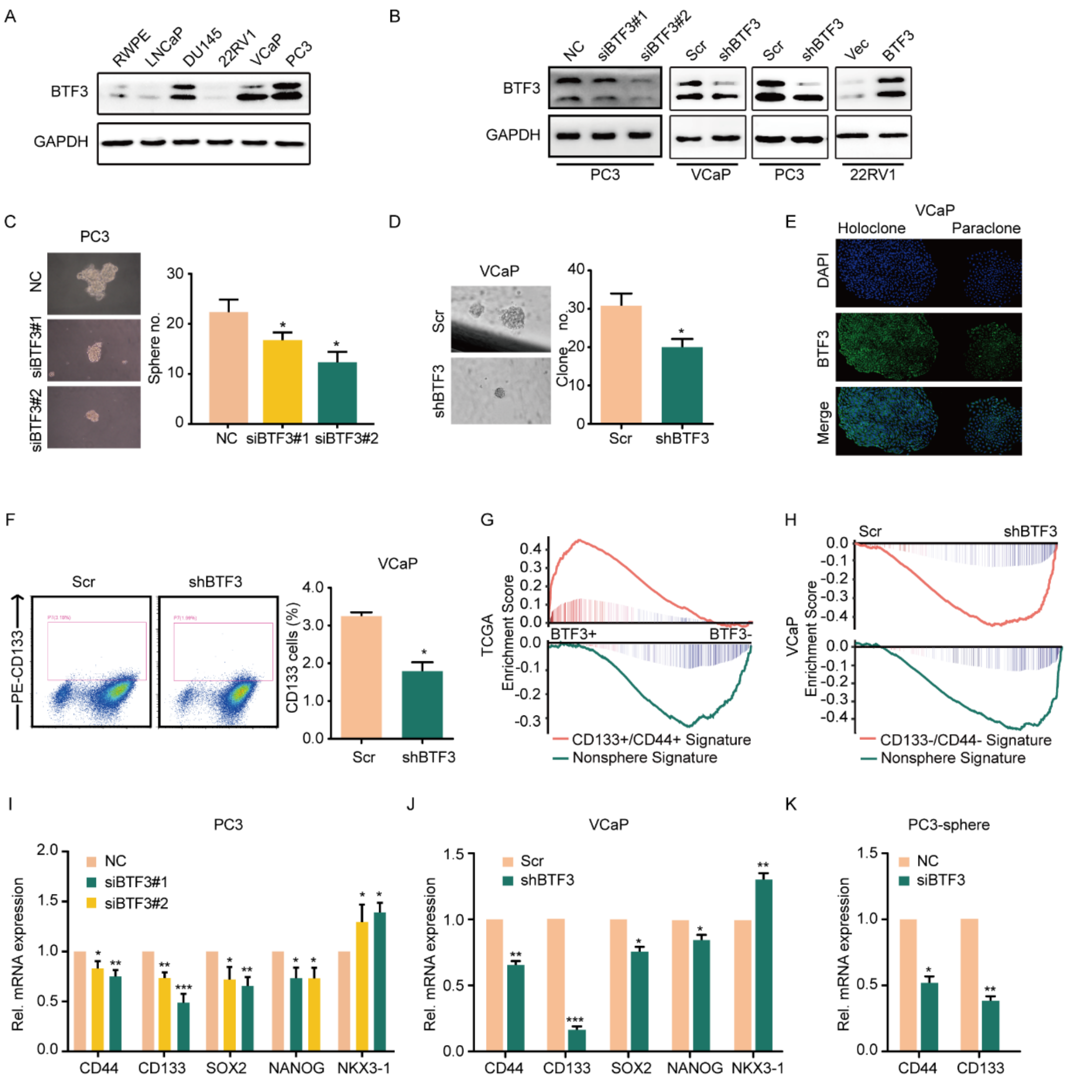

Figure S1 BTF3 overexpressed in PCa.

(A) Western blot analysis of BTF3 protein levels in human PCa cell lines.

(B) Western blot analysis of BTF3 protein levels in PCa cells. PC3 cells were transfected with two pairs of siRNAs targeting BTF3 (siBTF3) and negative control (NC). VCaP and PC3 cells were stably transfected with shRNA targeting BTF3 (shBTF3), negative control (Scr). 22RV1, LNCaP and RWPE cells were transfected with empty plasmids (Vec), plasmid expressing BTF3 (BTF3). The BTF3 levels in these established cell lines were verified by western blot after transfection.

(C) Sphere formation assay of PC3 cells. Left panel: Representative images of spheres.

Right panel: Quantitative results of sphereformation assays from triplicate experiments.

(D) Clonogenic assays in VCaP cells. Cells transfected as above were mixed with Matrigel and plated in 24-well plates and colonies counted in two weeks. Left panel: Representative images of clonogenic assay. Right panel: Quantitative results of clonogenic assay from triplicate experiments..

(E) Expression of BTF3 in holoclones and paraclones derived from VCaP cells by immunofluorescence analysis.

(F) Flow cytometry assay with CD133-PE antibody of VCaP Scr/shBTF3 cells. Left panel: FACS for CD133 indicated PCa cells. Right panel: Quantitative results of FACS from triplicate experiments.

(G) CD133+/CD44+ and nonsphere gene signatures in TCGA grouped by BTF3 expression with GSEA. (CD133+/CD44+ signature, ES=0.44, $P<0.001$, FDR $q<0.001$; nonsphere signature, ES=-0.35, $P=0.004$, FDR $q=0.004$).

(H) CD133-/CD44- and nonsphere gene signatures in VCaP Scr/shBTF3 microarray with GSEA. (CD133-/CD44- signature, ES=-0.45, $P<0.001$, FDR $q<0.001$; nonsphere signature, ES=-0.47, $P<0.001$, FDR $q<0.001$).

(I-K) The mRNA level of CSC markers, CD44, CD133, SOX2 and NANOG determined by real time PCR in PC3 cells (I), VCaP cells (J) and PC3 cells from second sphere formation (K) transfected as above.

* $P<0.05$, **$P<0.01$, ***$P<0.001$

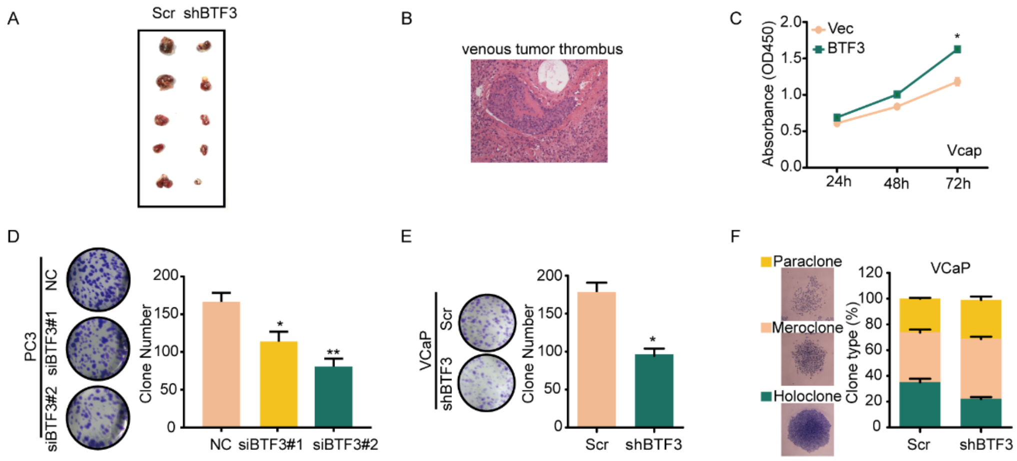

Figure S2 BTF3 functioned an oncogene in PCa

(A) Effect of BTF3 on turmorigenesis *in vivo* evaluated with xenografts model. PC3 cells with stable expression of Scr/shBTF3 subcutaneously injected into nude mice. Representative images of xenograft tumors were shown.

(B) Representative H&E images of xenograft tumor with venous tumor thrombus derived from PC3 shBTF3 cells. HE stain were performed to each tumor at harvest time.

(C) Cell viability as assessed by MTS assay of VCaP Vec/BTF3 cells.

(D-F) Clonal assay of PC3 cells and VCaP cells with BTF3 ablation. Cells were cultured for two weeks followed by staining with Giemsa and photography. Total number of all clones (D-E) and summary of three types of clones (F) presented as representative images (left) and quantification (right). * P<0.05, **P<0.01, ***P<0.001

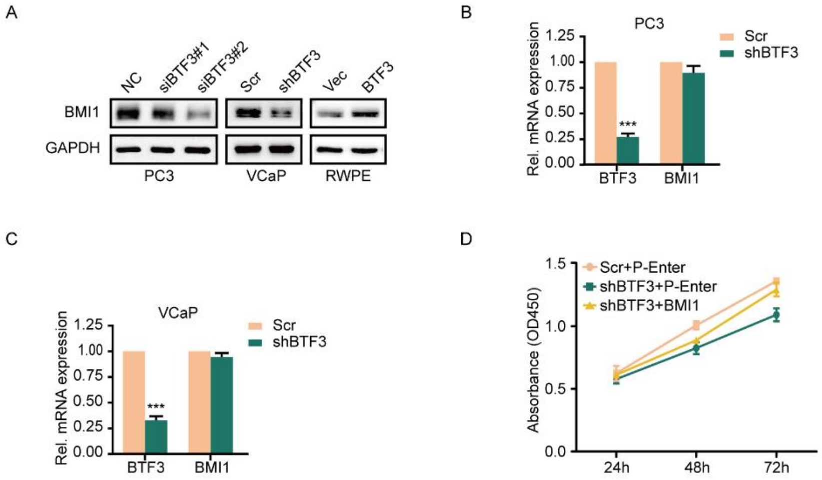

Figure S3 BTF3 targets BMI1 for stemness modeling of PCa cells.

(A) Western blot analysis of BTF3 and BMI1 expression in PCa cells with BTF3 knockdown or overexpression.

(B-C) The mRNA level of BTF3 and BMI1 were quantified by real time PCR in PC3(B) and VCaP cells (C) transfected with shRNA targeting BTF3 (shBTF3) or negative control (Scr).

(D) Effect of BMI1 in proliferation of BTF3. PC3 Scr/shBTF3 cells with were transiently transfected with empty plasmid (P-Enter) or BMI1 expression plasmid (BMI1) as indicated. Cells were subjected to MTS assay. * P<0.05, **P<0.01, ***P<0.001

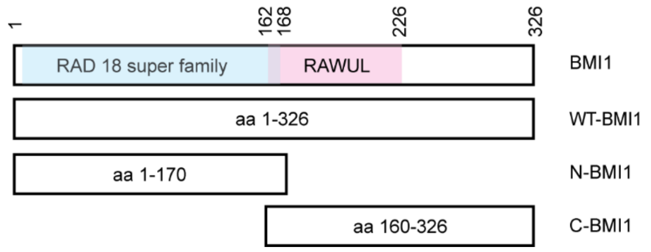

Figure S4

Schematic model of truncate BMI1 protein according to domains. The design of BMI1 constructs is based on known functional domains.

A

Subgroup	No. of Patients (%)	Event	Hazard Ratio (95% CI)	P
	217		2.40(1.42-4.06)	0.001
Risk	214			
Low	12 (5.5)	0		
Inter	37 (17.1)	6	2.65(0.476-14.728)	0.266
High	165 (76.0)	52	2.15(1.24-3.74)	0.007
Age	206			
<= 65	30 (14.5)	13	2.22(0.67-7.30)	0.191
> 65	176 (85.4)	42	2.69(1.45-5.00)	0.002
Pre PSA (ng/ml)	180			
<4	16 (8.9)	6	3.67(0.66-20.41)	0.138
4−10	22 (12.2)	7	12.72(1.46-110.49)	0.021
>10	142 (78.9)	33	2.08(1.05-4.14)	0.037
GS	214			
<7	29 (13.6)	3	1.06(0.87-12.77)	0.967
=7	76 (35.5)	14	3.48(1.17-10.40)	0.025
>7	109 (50.9)	41	1.87(1.00-3.49)	0.051
Stage	193			
T1−T2	147 (76.2)	29	2.71(1.28-5.75)	0.009
T3−T4	46 (23.8)	23	1.71(0.72-4.04)	0.221
Metastasis	191			
Negative	139 (72.8)	29	2.54(1.21-5.32)	0.013
Positive	52 (27.2)	23	1.83(0.75-4.47)	0.183

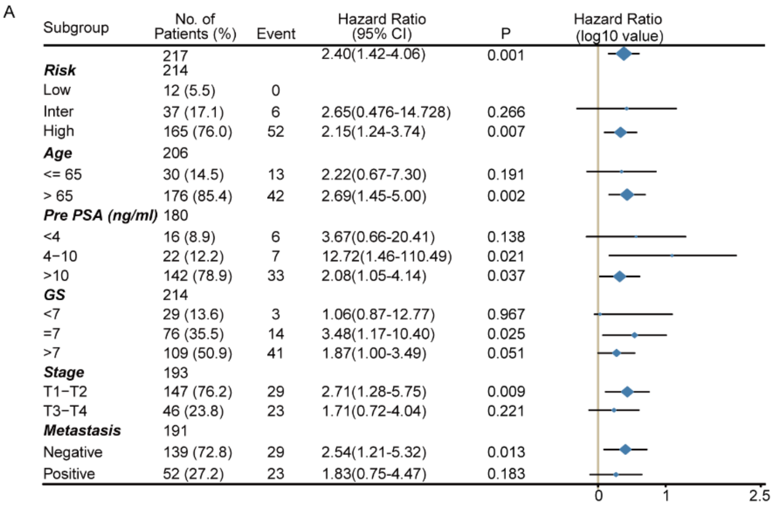

B

Subgroup	No. of Patients (%)	Event	Risk Ratio (95% CI)	P
	187 (100)	71	3.02(1.628-5.614)	<0.001
Risk	187			
Low	12 (6.4)	0		
Inter	30 (16)	8	4.50(0.77-26.13)	0.094
High	145 (77.5)	63	2.53(1.28-4.98)	0.008
Age	187			
<= 65	28 (15)	14	2.40(0.524-10.992)	0.259
> 65	159 (85)	57	3.09(1.56-6.10)	0.001
Pre PSA (ng/ml)	167			
<4	16 (9.6)	6	4.67(0.53-40.89)	0.164
4−10	19 (11.4)	6	11.00(1.14-106.43)	0.038
>10	132 (79)	45	2.51(1.19-5.32)	0.016
GS	187			
<7	29 (15.5)	5	0.95(0.09-10.50)	0.966
=7	65 (34.8)	16	2.77(0.86-8.90)	0.087
>7	93 (49.7)	50	2.86(1.22-6.69)	0.051
Stage	182			
T1−T2	139 (76.4)	39	3.32(1.53-7.19)	0.002
T3−T4	43 (23.6)	29	1.89(0.52-6.87)	0.334
Metastasis	180			
Negative	131 (72.8)	32	3.18(1.39-7.27)	0.006
Positive	49 (27.2)	36	2.24(0.61-8.21)	0.224

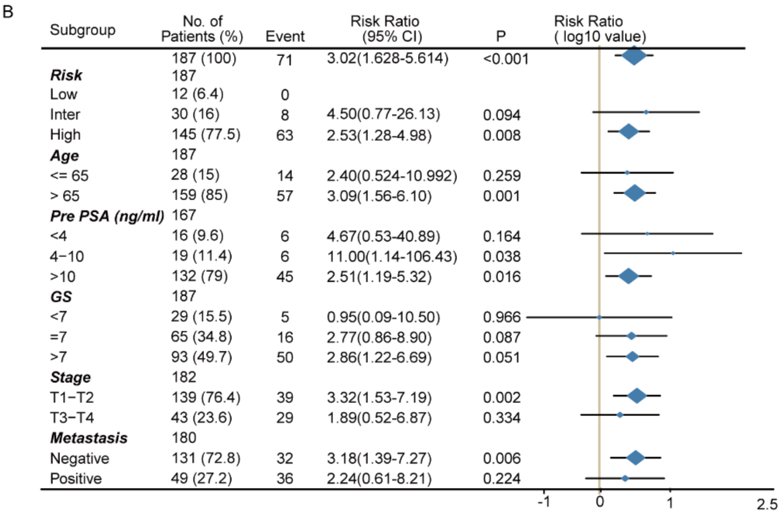

Figure S5

(A) Effect of BTF3 expression on overall survival in different subgroups.

(B) Effect of BTF3 expression on clinical recurrence in different subgroups.

GS= Gleason Score.

A

Prognositic models for overall survival of PCa patients	C-Index	bias corrected C-Index
Stage	0.65	0.65
Gleason Score	0.65	0.65
Pre PSA	0.51	0.51
Metastasis	0.59	0.59
Age	0.62	0.62
BTF3	0.61	0.61
Stage + Pre PSA + Gleason Score	0.69	0.67
Stage + Pre PSA + Gleason Score + BTF3	0.71	0.68
Stage + Pre PSA + Gleason Score + BTF3 + Metastasis	0.71	0.67
Stage + Pre PSA + Gleason Score + BTF3 + Age	0.74	0.70
Stage + Pre PSA + Gleason Score + BTF3 + Metastasis + Age	0.73	0.69

B

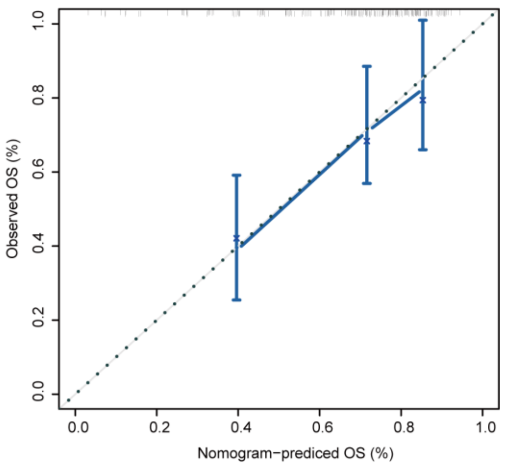

C

Subgroup	No. of Patients	Event	P	Hazard Ratio (95% CI)	AUC	P
	179	46	<0.0001	4.89 (2.19-10.93)	0.74	<0.001
Risk						
Low	12	0				
Inter	29	2	0.477	2.74 (0.17-43.91)		
High	138	44	0.002	3.80 (1.604-9.01)	0.67	0.002
Gleason Score						
<7	27	2	0.328	4.47 (0.22-89.76)		
=7	61	11	0.026	4.51 (1.19-17.04)	0.70	0.021
>7	91	33	0.031	4.86 (1.16-20.37)	0.65	0.025
Metastasis						
Negative	129	25	0.001	4.51 (1.80-11.33)	0.72	0.001
Positive	50	21	0.078	5.47 (0.85-40.80)		

Figure S6

(A) Comparison of accuracy of the prognostic models for overall survival.

(B) Plots depict the calibration of nomograms model in terms of agreement between predicted and observed 5-year outcomes. The x-axis represents the prediction calculated with use of the nomogram, and the y-axis represents the actual survival probability for our patients. Model performance is shown by the plot, relative to the 45-degree line, which represents perfect prediction.

(C) Cox proportional hazard regression analysis and AUC of the nomogram in subgroup of our PCa corhort.
