# Supplementary material for: BTF3 sustains cancer stem-like phenotype of prostate cancer via stabilization of BMI1
Source: J Exp Clin Cancer Res. 2019 May 28;38:227. doi: 10.1186/s13046-019-1222-z (PMC6540453; doi:10.1186/s13046-019-1222-z)
Supplement: Supplementary file 2 — Supplementary Materials and Methods. Cell lines and regents. Plasmid constructs and transfection. Clonal, clonogenic, and sphere-formation assays. Statistical analysis. (DOCX 17 kb) [file 13046_2019_1222_MOESM2_ESM.docx]

**Supplementary Materials and methods**

***Cell lines and regents***

Human prostate cancer cell lines (VCaP, PC3, 22RV1, DU145 and LNCaP), human prostate epithelial cell line (RWPE) and 293T(CRL-3216) were obtained from the American Type Culture Collection (ATCC, Virginia, USA) between 2012-2015 and authenticated again by short tandem repeat (STR) analysis again before and after our study. The cumulative culture length of the cells between thawing and use in this study was less than 15 passages. All of the newly revived cells were tested free of mycoplasma contamination by Hoechst 33258 staining (Beyotime, Jiangsu, China). The protein synthesis inhibitor cycloheximide (CHX) was purchased from Sigma-Aldrich. The proteasome inhibitor MG132 were purchased from MedChemExpress.

***Plasmid constructs and transfection***

Cells were transiently transfected with siRNAs and their corresponding control using Hiperfect transfection reagent (Qiagen). Plasmids were transiently transfected with Lipofectamine 2000 (Invitrogen, Carlsbad, CA, USA) following the manufacturer’s protocol. For stable knockdown of BTF3, Lenti-shBTF3-GFP, as well as its controls (Lenti-shSrc) were transfected into PCa cells. The targeted sequences for siRNAs and shRNA were described in **Supplementary Table 1**.

***Clonal, clonogenic, and sphere-formation assays***

For clonal experiments, cells were seeded at low density (500 cells/well) in a 6-well plate and allowed to grow until visible colonies appeared. Clones were counted within 2 weeks. For clonogenic assays, 100 μl of cells (1000 cells/well) was mixed with 100 μl of cold Matrigel and then plated around the rim of a 24-well dish. After solidification at 37°C for 15 min, 500 μl warm PrEBM was added in the center of the dish. Colonies were enumerated in 1–2 weeks. For sphere formation assay, 5000 single cells/well were cultured in serum-free DMEM/F12 (Thermo Fisher Scientific Cat#11330-032) supplemented with 1% B27 supplement (Thermo Fisher Scientific Cat#17504044), 1% N2 supplement (Thermo Fisher Scientific Cat#17502048), penicillin-streptomycin (100μg/ml; Thermo Fisher Scientific Cat#15140122), human recombinant epidermal growth factor (EGF; 20 ng/ml; R&D Systems Cat#236-EG-01M), and human recombinant basic fibroblast growth factor (bFGF; 10ng/ml; R&D Systems Cat#233-FB-025/CF) in ultralow attachment plate. For secondary sphere formation assay, the first spheres were trypsinized into single cells and re-seeded in the ultralow attachment plate. The second spheres with a diameter over 40μm were counted in 1-2 weeks after plating.

***Statistical analysis***

Statistical comparisons between groups were analyzed using two-sided Student’s t test and Mann-Whitney test. Correlation significance was assessed using χ^2^ test and Pearson's correlation coefficient test. Kaplan-Meier method and Cox regression model were used for to survival analysis. Cox regression coefficients to generate nomograms. C-Index and calibration plots were generated to explore the performance characteristics of the nomograms Nomogram, C-Index, and calibration plots were done with the rms package of R software, and all the other statistical tests were done with Graphpad prism 5 or SPSS 20.0 software. All statistical results from the quantitative analysis of the in vitro experiments are presented as means ±SEM or ±SD, as specified in the figure legends. p values < 0.05 were considered statistically significant.
